# Pituitary, Gonadal, Thyroid Hormones and Endocrine Disruptors in Pre and Postmenopausal Nigerian Women with ER-, PR- and HER-2-Positive and Negative Breast Cancers

**DOI:** 10.3390/medsci6020037

**Published:** 2018-05-18

**Authors:** Olulope Ajayi, Mabel Charles-Davies, John Anetor, Adeyinka Ademola

**Affiliations:** 1 Department of Chemical Pathology, College of Medicine, University of Ibadan, Ibadan 200212, Nigeria; mcharlesdavies@yahoo.com (M.C.-D.); johnanetor@gmail.com (J.A.); 2 Division of Surgical Oncology, Department of Surgery, University College Hospital, Ibadan 200212, Nigeria; deoluyinka@yahoo.com

**Keywords:** breast cancer, reproductive hormones, estrogen receptor, progesterone receptor, human epithelial receptor-2

## Abstract

Breast cancer is broadly sub-divided into hormone responsive and non-hormone responsive subtypes. Estradiol has been associated with hormone responsive breast cancers. There is, however, a paucity of information on the role of sex hormones, gonadotropins, and thyroid hormone in non-hormone responsive breast cancer. This study aimed to determine differences in the serum levels of sex hormones, gonadotropins, thyroid hormones, and endocrine disruptors (lead, cadmium, and arsenic) in Nigerian women with hormone responsive and non-hormone responsive breast cancers. Seventy-nine non-pregnant women aged 28–80 years with histologically confirmed breast cancer were recruited, pre-therapy, into this cross-sectional study. They comprised 52 premenopausal women and 27 postmenopausal women recruited from the Surgical Oncology Clinic of the Department of Surgery, University College Hospital, Ibadan. Comparison of biochemical parameters were based on the positivity (+) and negativity (−) of estrogen receptor (ER), progesterone receptor (PR) and human epithelial receptor-2 (HER-2). Estradiol, progesterone, follicle stimulating hormone (FSH), luteinizing hormone (LH), free thyroxine (FT_4_), free triiodothyronine (FT_3_), and thyroid stimulating hormone (TSH) were determined using enzyme immunoassay (EIA). Serum lead, cadmium and arsenic were determined using atomic absorption spectrophotometry (AAS). Expression of ER, PR and HER2 were determined using immunohistochemistry. Data was analyzed using Mann-Whitney *U*-test and multiple regression, with *p* < 0.05 considered as being statistically significant. Estradiol and progesterone were significantly higher in breast cancer participants with ER^−^ and PR^−^ compared with those with ER^+^ and PR^+^ breast cancer (*p* < 0.05). Follicle stimulating hormone and LH levels were significantly higher in participants with ER^+^ and PR^+^ breast cancer compared with participants with ER^−^ and PR^−^ breast cancer (*p* < 0.05). Arsenic was inversely related with TSH in premenopausal participants with ER^−^ and PR^−^ (*β* = −0.305; *β* = −0.304, respectively). Sex hormones and gonadotropins appear to be involved in the pathogenesis of triple negative and luminal breast cancer, respectively.

## 1. Introduction

Breast cancer is a multifaceted disease with distinct biological subtypes. It presents with a varied spectrum of clinical, pathologic, and molecular features, with different prognostic and therapeutic implications [1]. Clinicians rely on traditional clinico-pathological, and readily available, tumor markers such as estrogen receptor (ER), progesterone receptor (PR), and human epithelial receptor-2 (HER-2) because they are reliable, inexpensive, and useful for therapeutic decision making. This makes them a reasonable substitute for other, more expensive, molecular sub-typing tools [2,3]. These receptors provide a means by which specific ligands bind resulting in transformation of the inactive oligomeric complex into an active state that regulates gene expression [4,5].

Hormones are considered to be important in the etiology of breast cancer. High levels of estrogens have been associated with breast carcinogenesis [6]. Alkylation of cellular molecules, generation of active radicals, and genotoxicity of estrogen metabolites are reportedly involved in the initiation, promotion, and progression of breast cancer [7,8,9].

The role of progesterone in breast cancer is controversial. It is thought that it increases breast cancer risk in premenopausal women at the luteal phase. Breast mitotic rates are highest in the luteal phase of the menstrual cycle [6,10]. Follicle stimulating hormone (FSH) stimulates follicle growth and development in the ovaries and has been associated with certain cancers including breast cancer [11]. Some reports showed that FSH induces cancer cell proliferation, differentiation, and metastasis by activating adenylyl cyclase, which results in increased cyclic adenosine monophosphate(cAMP) levels [12,13]. There is currently a paucity of information on the association of gonadotropins with hormone receptor-positive and negative breast cancer in women with breast cancer in Sub-Saharan Africa. Furthermore, the relationship between thyroid hormones and breast cancer is not clearly defined. Some studies observed that thyroid diseases are common in women with breast cancer, while some had contrary reports [14,15].

Toxic metals, such as lead, cadmium, and arsenic, are a major source of oxidative stress, which is implicated in the development of breast cancer [16]. The differential roles of the toxic metals in the pathogenesis and course of hormone responsive and non-hormone responsive breast cancers are poorly understood. At present, information on the serum levels of pituitary, gonadal, thyroid hormones, and endocrine disruptors in Nigerian women with ER-, PR-, HER-2-positive (+) and negative (−) breast cancers are sparse. Therefore, this study was carried out to compare serum levels of pituitary, gonadal, and thyroid hormones in premenopausal and postmenopausal Nigerian breast cancer participants with positive and negative ER, PR and HER-2.

## 2. Materials and Methods

### 2.1. Study Design and Participants

The study was a cross-sectional study. The study protocol was approved by the University of Ibadan and University College Hospital Health Review Committee (UI/EC/10/0193). Informed consent was obtained from the participants before their recruitment. 

Seventy-nine non-pregnant women with histologically confirmed breast cancer were recruited into this study, pre-therapy. Enrolment and basic characteristics of the study participants have previously been reported [11]. Participants were recruited from the Surgical Oncology Clinic of the Department of Surgery, University College Hospital, Ibadan, by a consultant surgical oncologist. Women who reported being on hormonal drugs (i.e., contraceptives and hormone replacement therapy), or had other types of malignancies or chronic diseases, were excluded from the study. Participants were reported as postmenopausal if they were 55 years and older and had stopped menstruating over the last 12months. Participants with bilateral oophorectomy were considered as postmenopausal

The study participants were then grouped based on their immunohistochemistry results i.e., 69 ER^−^ versus 10 ER^+^; 71 PR^−^ versus 8 PR^+^; 64 HER-2^−^ versus 15 HER-2^+^; 17 postmenopausal ER^−^ versus 10 postmenopausal ER^+^; 19 postmenopausal PR^−^ versus 8 postmenopausal PR^+^; 46 premenopausal HER-2^−^ versus 6 premenopausal HER 2^+^; and 18 postmenopausal HER-2^−^ versus 9 postmenopausal HER-2^+^.

All the premenopausal participants in the study were ER- and PR-negative. Of the study participants, 83.5% presented at advanced stages of the disease (stages 3 and 4), while 16.5% of the study participants presented at stages 1 and 2 [11].

### 2.2. Sample Collection

Ten milliliters of venous blood were aseptically obtained by venipuncture from each participant and dispensed into a plain bottle. For premenopausal participants, blood samples were drawn between days five and nine of their menstrual cycle in the follicular phase (forward dating) and five to nine days before the anticipated start of their next menstrual cycle in the luteal phase (backward dating) [10]. All samples were allowed to retract and were centrifuged at 3500 rpm for five minutes. The serum obtained was aspirated into clean vials and stored at −20 °C until analyses were done.

### 2.3. Hormonal Assay and Toxic Metals Estimation

Serum progesterone, estradiol (E_2_), luteinizing hormone (LH), FSH, free triiodothyronine (FT_3_), free thyroxine (FT_4_), and thyroid stimulating hormone (TSH)were determined using enzyme immune assay (EIA) on TOSOH AIA System Analyzers (Tosoh Corporation, Tokyo, Japan). Serum arsenic, cadmium, and lead were determined using atomic absorption spectrophotometry (210 VGP Atomic Absorption Spectrophotometer, Buck Scientific, Norwalk, CT, USA).

### 2.4. Statistical Analysis

Data was analyzed using Statistical Package for Social Scientists (SPSS) software, version 18 (IBM, New York, NY, USA). Mann Whitney *U*-test was used for the comparison of the non-parametric variables. Multiple regression was used to determine the relationship between variables, with *p*-values less than 0.05 being considered as statistically significant. 

## 3. Results

Table 1 shows the serum levels of selected hormones and toxic metals in ER^−^ and ER^+^ breast cancer participants. Estradiol and progesterone were significantly higher in participants with ER^−^ compared with participants with ER^+^. Moreover, estradiol was significantly higher in postmenopausal participants with ER^−^ compared with postmenopausal participants with ER^+^. Luteinizing hormone and FSH were significantly higher in participants with ER^+^ compared with participants with ER^−^. Follicle stimulating hormone was also significantly higher in postmenopausal participants with ER^+^ compared with participants with ER^−^.

As shown in Table 2, estradiol and progesterone were significantly higher in participants with PR^−^ compared with women with PR^+^. Moreover, estradiol was significantly higher in postmenopausal participants with PR^−^ compared with postmenopausal participants with PR^+^. Luteinizing hormone and FSH were significantly higher in participants with PR^+^ compared with participants with ER^−^. Follicle stimulating hormone was also significantly higher in postmenopausal participants with PR^+^ compared with participants with PR^−^.

Progesterone was significantly higher in participants with HER-2^−^ compared with participants with HER-2^+^, while FSH was significantly higher in participants with ER+ compared with participants with HER-2^−^ (Table 3).

Table 4 shows the multiple regression of variables. In premenopausal women with ER^−^, PR^−^, and HER-2^−^, progesterone was positively related with FT_3_, but inversely related with TSH (*β* = 0.246, *β* = −0.272; *β* = 0.229, *β* = −0.301; *β* = 0.650, *β* = −0.677, respectively). In premenopausal women with ER^−^ and PR^−^, estradiol was positively related with progesterone and TSH (*β* = 0.724, *β* = 0.260; *β* = 0.843, *β* = 0.120, respectively). Moreover, progesterone was inversely related with arsenic (*β* = −0.216; *β* = −0.188). Thyroid stimulating hormone was also inversely related with arsenic (*β* = −0.330; *β* = −0.304, respectively). In premenopausal women with ER^−^, FT_4_ was inversely related with arsenic (*β* = −0.330), while TSH was positively related with FSH (*β* = 0.281). In postmenopausal women with ER^−^, PR^−^, and HER-2^−^, LH was inversely related with lead (*β* = −4.843; *β* = −4.843; *β* = −6.995, respectively), while LH was positively related with cadmium (*β* = 5.045; *β* = 5.045; *β* = 6.803). In postmenopausal women with HER-2^−^, FSH was positively related with LH (*β* = 0.963), while progesterone was positively related with E_2_ (*β* = 0.896).

## 4. Discussion

Breast cancer is a heterogeneous disease with a wide spectrum of clinical, pathologic, and molecular features [17]. It comprises luminal, HER-2^+^, and triple negative subtypes [18]. Luminal and HER-2^+^ breast cancers are responsive to endocrine and anti-HER-2 therapies, respectively. However, chemotherapy is effective in the management of triple negative breast cancer [19].

Premenopausal breast cancer is a challenge to patients, their family members, and the health care system. It is associated with adverse pathological factors, such as high-grade tumor, presentation at a later stage than their older counterparts, and hormone receptor negativity [20]. The premenopausal women in this study were all ER^−^ and PR^−^. This is similar to the findings of Makanjuola et al. [21]. The adverse pathological factors that characterize triple negative breast cancer could be the basis of their worse disease outcome. Women with triple negative breast cancer (ER^−^, PR^−^ and HER-2^−^) in this study may benefit from chemotherapy as the first line of treatment. This is because they lack the appropriate hormone receptors. Participants with luminal breast cancer can, however, benefit from endocrine therapy.

High levels of endogenous steroid hormones, particularly estrogens, are believed to increase breast cancer risk [6]. In this study, estradiol was significantly higher in in breast cancer participants with negative ER and PR expressions, as compared with participants with positive ER and PR expression. A similar observation was also made in the postmenopausal participants in this study. This contrasts with the report of Farhat et al. [22], which showed that there is higher level of estradiol in postmenopausal women with ER+ breast cancer. However, Kim et al. [23] reported a similar observation to our study. They observed that serum estradiol level adversely affect prognosis in patients with ER-negative tumors. Estrogen mediates important cellular activity in estrogen receptor-negative breast cancer cells via membrane associated G protein-coupled receptor 30 (GPR30). This is a non-ER-mediated pathway involving ezrin-dependent cytoskeleton rearrangement, which elicits a stimulatory effect on cell migration and invasion [24]. Gupta et al. [25] observed that estradiol enhanced tumor formation of weakly tumorigenic ER^−^ human mammary epithelial cells (HMLE-Ras) when injected into the mammary fat pad of laboratory animals. This effect was caused by the systemic induction of angiogenesis and stromal cell recruitment by estradiol. These observations, together with ours, suggest that estradiol can act as a potent promoter of metastasis in ER^−^ tumors through mechanisms involving the host microenvironment.

The role of estrogen in breast cancer as a potent breast mitogen is undisputed, however, the role of progesterone is grossly understudied [26]. In this study, progesterone was significantly higher in breast cancer participants with ER^−^, PR^−^, and HER-2^−^ (triple negative) breast cancer compared with participants with positive ER, PR, and HER-2 expressions. Primarily, progesterone may act via protooncogenes and growth factors to affect breast cell proliferation and breast cancer etiology [26]. The absence of the appropriate receptors for the hormones could cause the loss of feedback mechanisms in the women with triple negative breast cancer. 

Pituitary production of LH and FSH in premenopausal women is controlled by the secretion of gonadotropin releasing hormone (GnRH) from the hypothalamus. After menopause, the pulsatility in gonadotropin secretion is a consequence of a reduced release of hypothalamic GnRH and of a decreased sensitivity of the pituitary gonadotropin [27]. Previous research has linked gonadotropins with breast cancer [28]. In this study, serum LH and FSH levels were significantly higher in participants with positive ER and PR expressions compared with participants who were ER- and PR-negative, respectively. Increased FSH levels were also observed in postmenopausal women with ER^+^ and PR^+^ compared with postmenopausal women with ER^−^ and PR^−^ in this study. Follicle stimulating hormone increases with age and correlates with a loss of ovarian reserve [29]. Our observation suggests that gonadotropins could be involved in the pathogenesis of hormone responsive breast cancers. Tanaka et al. [30] observed that gonadotropin acts on breast cancer cells and accelerates the conversion of dehydroepiandrosterone (DHEA) into estrogens, thereby, stimulating the growth of estrogen-dependent tumorous cells.

Follicle stimulating hormone levels were significantly higher in women with HER-2^+^ breast cancer compared with HER-2^−^ breast cancer in this study. This aligns with the reports of Zhou et al. [31]. Human epithelial receptor-2-positive breast cancer grows faster, spreads rapidly, has increased risk of loco-regional recurrence, and increases breast cancer mortality [32,33]. Observations from this study indicate that FSH could be involved in the proliferation of HER-2^+^ breast cancer. This could be via mechanisms involving tyrosine kinase, which drives the proliferation of cells and evasion of apoptosis, genetic, and epigenetic modifications, as well as alterations in upstream regulators, such as the fox headbox p3 (Fox p3) and GATA4 [34].

Changes in thyroid hormone levels have been associated with proliferative activity of breast cancer [35]. In this study, there was no difference in the thyroid hormone levels in the premenopausal and postmenopausal women with positive and negative hormone receptors. However, a positive relationship was observed between E_2_ and TSH in premenopausal participants who were ER- and PR-negative. The biological activity of thyroid hormones and estradiol manifests in cells expressing their respective receptors, which belong to the nuclear receptor family. Moreover, estrogen-like effects of thyroid hormones have been reported [36]. Progesterone was positively related with FT_3_, but inversely related with TSH in premenopausal participants with triple negative breast cancer. This relationship was not observed in postmenopausal participants. The reason for this observation is not clear, but suggests an interplay between progesterone and thyroid hormones in the pathogenesis of non-hormone responsive breast cancer.

Studies have suggested an association between exposure to heavy metals and carcinogenesis [37,38]. In this study, there were no differences in the serum lead, cadmium, and arsenic between women with positive hormone receptors and negative hormone receptors. However, an inverse relationship was observed between TSH and arsenic in premenopausal women with ER^−^ and PR^−^ breast cancer. Arsenic is a potent endocrine disruptor, which has been associated with malignancies. Davey et al. [39] observed an interference of thyroid hormone metabolism by arsenic. One hypothesized mechanism of thyroid hormone disruption is through the affection of the thyroid receptor (TR) response elements and the expression of type 1 *deiodinase* gene [40]. In addition, the inverse relationship of progesterone with arsenic also suggests the possible interference of progesterone metabolism by arsenic, which could result in breast carcinogenesis.

Lead and cadmium can cross the blood-brain barrier and, thereby, disrupt the hypothalamic-pituitary axis [41]. In postmenopausal women with ER^−^ and PR^−^, a positive relationship was observed between cadmium and LH. This buttresses the endocrine disrupting property of Cd, although, the mechanism through which this is carried out is poorly understood at present.

## 5. Conclusions

Findings from this study suggest that estradiol and progesterone may be involved in the pathogenesis of triple negative breast cancer, while gonadotropins may be involved in luminal breast carcinogenesis

## 6. Study Limitation

Small sample size was a limitation in this study, therefore, studies with a large sample size are suggested to substantiate our findings.

## Figures and Tables

**Table 1:** Serum levels of hormones and toxicants in ER-negative and ER-positive breast cancer participants.

Parameter	ER^−^ (*n* = 69)	ER^+^ (*n* = 10)	Postmeno ER^−^ (*n* = 17)	Postmeno ER^+^ (*n* = 10)	*p_1_*-value	*p_2_*-value
E_2_ (pmol/L)	295.70 (170.71–480.33)	98.66 (82.29–220.79)	140.68 (120.60–236.79)	95.42 (81.16–193.97)	0.001 *	0.038 *
Prog (nmol/L)	2.08 (1.33–7.33)	1.29 (0.83–1.74)	1.54 (1.11–2.50)	1.19 (0.82–1.63)	0.010 *	0.153
LH (IU/L)	8.70 (4.30–16.40)	24.75 (19.60–33.20)	19.20 (15.60–34.80)	26.10 (18.60–34.20)	0.000 *	0.225
FSH (IU/L)	8.20 (5.18–18.26)	77.77 (53.55–98.85)	43.70 (29.75–58.30)	78.14 (54.20–103.50)	0.000 *	0.038 *
FT_3_ (pmol/L)	3.18 (2.75–3.53)	3.30 (2.81–3.60)	3.12 (2.50–3.54)	3.31 (2.79–3.65)	0.585	0.666
FT_4_ (pmol/L)	16.96 (14.94–20.36)	16.38 (15.35–20.26)	17.95 (15.83–20.28)	15.84 (15.17–20.48)	0.863	0.535
TSH (mIU/L)	1.43 (0.86–2.37)	1.12 (0.83–1.45)	1.54 (1.07–1.88)	1.16 (0.79–1.70)	0.381	0.269
Pb (µg/dL)	5.22 (4.13–6.46)	5.75 (5.54–6.88)	5.22 (4.21–6.32)	5.82 (5.40–7.07)	0.143	0.112
Cd (µg/dL)	0.04 (0.03–0.05)	0.04(0.04–0.05)	0.04 (0.03–0.05)	0.05 (0.04–0.06)	0.284	0.222
As (µg/dL)	0.27 (0.24–0.36)	0.36(0.22–0.42)	0.30 (0.25–0.35)	0.40 (0.20–0.44)	0.416	0.808

Values are in median (interquartile range); *n*, number of subjects; ER^−^, women with estrogen receptor negative; ER^+^, women with estrogen receptor positive; Postmeno ER^−^, postmenopausal women with estrogen receptor negative; Postmeno ER^+^, postmenopausal women with estrogen receptor positive; Prog, progesterone; E_2_, estradiol; LH, luteinizing hormone; FSH, follicle stimulating hormone; FT_3_, free triiodothyronine; FT_4_, free thyroxine; TSH, thyroid stimulating hormone; IU, international units; *p_1_*-value, probability between women with ER^−^ and ER^+^, *p_2_*-value, probability between postmenopausal women with ER-negative and ER-positive, * significant at *p* < 0.05.

**Table 2:** Serum levels of hormones and toxicants in PR-negative and PR-positive breast cancer participants.

Parameter	PR^−^ (*n* = 71)	PR^+^ (*n* = 8)	Postmeno PR^−^ (*n* = 19)	Postmeno PR^+^ (*n* = 8)	*p_1_*-value1	*p_2_*-value
E_2_ (pmol/L)	290.53 (169.77–477.70)	95.42 (81.16–193.97)	140.68 (120.60–236.79)	95.42 (81.16–193.97)	0.000 *	0.038 *
Prog (nmol/L)	2.06 (1.31–5.91)	1.19 (0.82–1.63)	1.54 (1.11–2.50)	1.19 (0.82–1.63)	0.010 *	0.153
LH (IU/L)	8.70 (4.20–15.90)	26.10 (18.60–34.20)	19.20 (15.60–34.80)	26.10 (18.60–34.20)	0.000 *	0.225
FSH (IU/L)	8.30 (5.24–19.08)	78.14 (54.20–103.50)	43.70 (29.75–58.30)	78.14 (54.20–103.50)	0.000 *	0.038 *
FT_3_ (pmol/L)	3.14 (2.74–3.49)	3.31 (2.79–3.65)	3.12 (2.50–3.54)	3.31 (2.79–3.65)	0.506	0.666
FT_4_ (pmol/L)	16.96 (14.87–20.01)	15.84 (15.17–20.48)	17.95 (15.83–20.28)	15.84 (15.17–20.48)	0.808	0.535
TSH (mIU/L)	1.47 (0.88–2.39)	1.16 (0.77–1.70)	1.54 (1.07–1.88)	1.16 (0.79–1.70)	0.385	0.269
Pb (µg/dL)	5.22 (4.13–6.54)	5.82 (5.40–7.07)	5.22 (4.21–6.33)	5.82 (5.40–7.07)	0.152	0.112
Cd (µg/dL)	0.04 (0.03–0.05)	0.05 (0.04–0.06)	0.04 (0.03–0.05)	0.05 (0.04–0.06)	0.280	0.222
As (µg/dL)	0.27 (0.24–0.36)	0.4 (0.20–0.44)	0.30 (0.25–0.35)	0.36 (0.20–0.44)	0.583	0.808

Values are in median (interquartile range); *n*, number of subjects; PR^−^, women with progesterone receptor negative; PR^+^, women with progesterone receptor-positive; Postmeno PR^−^, postmenopausal women with progesterone receptor-negative; Postmeno PR^+^, postmenopausal women with progesterone receptor-positive; Prog, progesterone, E_2_, estradiol; LH, luteinizing hormone; FSH, follicle stimulating hormone; FT_3_, free triiodothyronine; FT_4_, free thyroxine; TSH, thyroid stimulating hormone. *p_1_*-value, probability between women with PR^−^ and PR^+^, *p_2_*-value, probability between postmenopausal women with PR^−^ and PR^+^, * significant at *p* < 0.05.

**Table 3:** Serum levels of hormones and toxicants in HER-2- negative and HER-2 positive breast cancer participants.

Parameter	HER-2^−^ (*n* = 64)	HER-2^+^ (*n* = 15)	*p*-value
E_2_ (pmol/L)	251.80 (147.30–463.05)	219.64 (136.42–376.33)	0.803
Prog (nmol/L)	1.80 (1.33–6.06)	1.45 (0.83–2.69)	0.049*
LH (IU/L)	9.33 (4.60–16.78)	16.60 (7.20–22.00)	0.179
FSH (IU/L)	8.35 (5.35–22.85)	35.20 (10.03–77.40)	0.010*
FT_3_ (pmol/L)	3.19 (2.80–3.53)	2.75 (2.44–3.45)	0.171
FT_4_ (pmol/L)	17.05 (14.96–20.85)	16.64 (14.79–17.77)	0.255
TSH (mIU/L)	1.50 (0.88–2.35)	1.16 (0.85–1.63)	0.446
Pb (µg/dL)	5.53 (4.21–6.62)	5.62 (3.70–5.99)	0.465
Cd (µg/dL)	0.04 (0.03–0.05)	0.04 (0.03–0.05)	0.335
As (µg/dL)	0.30 (0.24–0.36)	0.24 (0.24–0.34)	0.384

Values are in median (interquartile range); *n*, number of subjects; HER-2^−^, women with human epithelial receptor-2-negative; HER^+^, women with human epithelial receptor-2-positive; Postmeno HER-2^−^, postmenopausal women with human epithelial receptor-2-negative; Postmeno HER-2+, postmenopausal women with human epithelial receptor-2-positive; Prog, progesterone; E_2_, estradiol; LH, luteinizing hormone; FSH, follicle stimulating hormone; FT_3_, free triiodothyronine; FT_4_, free thyroxine; TSH, thyroid stimulating hormone; *p*-value, probability between premenopausal women with HER-2^−^ and HER-2^+^; * significant at *p* < 0.05.

**Table 4:** Multiple regression analysis of variables in pre and postmenopausal women with ER-, PR-and HER2-negative breast cancer.

Group	Dependent	Predictors	*β*	*p*-Value
Premenopausal ER^−^				
*R^2^* = 0.611, F = 7.686, *p* = 0.000	E_2_	Prog	0.724	0.000
		TSH	0.260	0.017
*R^2^* = 0.639, F = 8.645, *p* = 0.000	Prog	FT_3_	0.246	0.013
		TSH	−0.272	0.009
		E_2_	0.673	0.000
		As	−0.216	0.033
*R^2^* = 0.138, *F* = 0.781, *p* = 0.635	FT_4_	As	−0.330	0.035
*R^2^* = 0.296, *F* = 2.058, *p* = 0.055	TSH	As	−0.305	0.030
		FSH	0.281	0.037
Postmenopausal ER^−^				
*R*^2^ = 0.894, *F* = 6.589, *p* = 0.011	LH	Pb	−4.843	0.003
		Cd	5.045	0.004
Premenopausal PR^−^				
*R*^2^ = 0.693, *F* = 10.547, *p* = 0.000	E_2_	TSH	0.320	0.002
		Prog	0.843	0.000
*R*^2^ = 0.227, *F* = 1.374, *p* = 0.231	FT_3_	Prog	0.655	0.010
*R*^2^ = 0.354, *F* = 2.558, *p* = 0.019	TSH	As	−0.304	0.028
		Prog	−0.728	0.001
		E_2_	0.673	0.002
*R*^2^ = 0.730, *F* = 12.637, *p* = 0.000	Prog	As	−0.188	0.036
		E_2_	0.741	0.000
		FT_3_	0.229	0.010
		TSH	−0.301	0.001
Postmenopausal PR^−^				
	LH	Pb	−4.843	0.003
		Cd	5.045	0.004
Premenopausal HER-2^−^				
*R*^2^ = 0.284, *F* = 1.498, *p* = 0.188	FT_3_	Prog	0.650	0.009
*R*^2^ = 0.329, *F* = 1.848, *p* = 0.095	TSH	Prog	−0.677	0.005
		E_2_	0.685	0.003
Postmenopausal HER-2^−^				
*R*^2^ = 0.839, *F* = 5.777, *p* = 0.006	LH	Pb	−6.995	0.000
		Cd	6.803	0.000
		FSH	0.483	0.015
*R*^2^ = 0.883, *F* = 8.418, *p* = 0.001	Prog	E_2_	0.896	0.000
*R*^2^ = 0.679, *F* = 2.346, *p* = 0.100	FSH	LH	0.963	0.015

*B*, Beta coefficient; *F*, *F*-statistics; *p*-value, probability; E_2_, estradiol; LH, luteinizing hormone; FT_3_, triiodothyronine; FSH, follicle stimulating hormone, TSH, thyroid stimulating hormone; ER, estrogen receptor; PR, progesterone receptor; HER-2, human epithelial receptor-2.

## References

[1] Onitilo A., Engel J.M., Greenlee T.R., Mukesh B.N. (2009). Breast cancer subtypes based on estrogen receptor, progesterone receptor and human epithelial receptor 2 expression. Comparison of clinicopathologic features and survival. Clin. Med. Res..

[2] Badve S.S., Baehner F.L., Gray R.P., Childs B.H., Maddala T., Liu M.L., Rowley S.C., Shak S., Perez E.A., Shulman L.J. (2008). Estrogen and progesterone receptors status in ECOG 2197; Comparison of immunohistochemical by local and central laboratories and quantitative reverse transcription polymerase chain reaction by central Laboratory. J. Clin. Oncol..

[3] Brown M., Tsodikov A., Bauer K.R., Parise C.A., Caggiano V. (2008). The role of human epithelial growth factor receptor 2 in the survival of women with estrogen and progesterone receptor negative, invasive breast cancer. The California cancer registry 1999–2004. Cancer.

[4] Umayahara Y., Kawamori R., Watada H., Imano E., Iwama N., Morishima T., Yamasaki Y., Kajimoto Y., Kamada T. (1994). Estrogen regulation of the insulin-like growth factor 1 gene transcription involves and AP-1 enhancer. J. Biol. Chem..

[5] Horwitz K.B., Jackson T.A., Bain D.L., Richer J.K., Takimoto G.S., Tung L. (1996). Nuclear receptor co-activators and co-repressors. Mol. Endocrinol..

[6] Ho C.C.K., Rohaizak M., Zulkifli S.Z., Siti-Aishah M.A., Nor-Aini U., Sharifah-Noor-Akmal S.H. (2009). Serum sex hormone levels in pre and postmenopausal breast cancer patients. Singap. Med. J..

[7] Russo I.H., Russo J. (1998). Role of hormones in mammary cancer initiation and progression. J. Mammary Gland Biol. Neoplast..

[8] Brisken C. (2008). Endocrine disruptors and breast cancer. Chimia.

[9] Yager J.D., Davidson N.E. (2006). Mechanisms of disease: Estrogen carcinogenesis in breast cancer. N. Engl. J. Med..

[10] Wang B., Mi M., Wang J., Wei N., Zhang Q., Zhu J., Yang S., Guo B., Xu J., Yang X. (2009). Does the increase of endogenous steroid hormone levels also affect breast cancer risk in Chinese women? A case-control study in Chongqing, China. Int. J. Cancer.

[11] Ajayi O., Charles-Davies M., Anetor J., Adeyinka A. (2016). Sex hormones, oestrogen receptor, progesterone receptor and human epithelial receptor 2 expressions in pre and postmenopausal sub-Saharan African women with breast cancer. J. Cancer Tumour Int..

[12] Tunizicker-Dun M., Maizels E.T. (2006). FSH signaling pathways in immature granulose cells that regulate target gene expression; branching out from protein kinase A. Cell Signal..

[13] Fan H.Y., Cheng X., Richards J.S. (2007). FSH induces multiple signaling cascades; evidence that activation of Rous sarcoma oncogene RAS and epidermal growth factor receptor are critical for differentiation. Mol. Endocrinol..

[14] Tosovic A., Bondesson A.G., Bonseson L., Ericsson U.B., Malm J., Manjer J. (2010). Prospectively measured triiodothyronine levels are positively associated with breast cancer risk in postmenopausal women. Breast Cancer Res..

[15] Tosovic A., Becker C., Bondeson A.G., Ericsson U.B., Malm J., Manjer J. (2012). Prospectively measured thyroid hormone and thyroid peroxidase antibodies in relation to breast cancer risk. Int. J. Cancer.

[16] Rossner P., Gammon M.D., Terry M.B., Agrawal M., Zhang F.F., Teitelbaum S.L., Eng S.M., Gaudet M.M., Neugut A.I., Santella R.M. (2006). Relationship between urinary 15-F2t- Isoprostane and 8-Oxodeoxyguanosine levels and breast cancer risk. Cancer Epidemiol. Biomark. Prev..

[17] Parise C.A., Caggiano V. (2014). Breast cancer survival defined by estrogen receptor, progesterone receptor and human epithelial receptor 2 subtypes and a surrogate classification according to tumour grade and immunohistochemical biomarkers. J. Cancer Epidemiol..

[18] Perou C.M., Sorlie T., Eisen M.B., van de Rijn M., Jeffrey S.S., Rees C.A., Pollack J.R., Ross D.T., Johnsen H., Akslen L.A. (2000). Molecular portraits of human breast tumors. Nature.

[19] Cardoso F., Costa A., Senkus E., Aapro M., Andre F., Barrios C.H., Bergh J., Bhattacharyya G., Biganzoli L., Cardoso M.J. (2017). 3rd ESO–ESMO International Consensus Guidelines for Advanced Breast Cancer (ABC 3). Ann. Oncol..

[20] Assi H.A., Khoury K.E., Dbouk H., Khalil L.E., Mouhieddine T.H., El Saghir N.S. (2013). Epidemiology and prognosis of breast cancer in young women. J. Thorac. Dis..

[21] Makanjuola S.B.L., Ayodele S.D., Javid F.A., Obafunwa J.O., Oludara M.A., Popoola A.O. (2014). Breast cancer receptor status assessment and clinicopathological association in Nigerian women: A retrospective analysis. J. Cancer Res. Therapy.

[22] Farhat G.N., Cummings S.R., Chlebowski R.T., Parimi N., Cauley J.A., Rohan T.E., Huang A.J., Vitolins M., Hubbell F.A., Manson J.E. (2011). Sex hormone levels and risk of estrogen receptor negative and estrogen positive breast cancer. J. Natl. Cancer Inst..

[23] Kim J.Y., Han W., Moon H.G., Ahn S.K., Kim J., Lee J.W., Kim M.K., Kim T., Noh D.Y. (2013). Prognostic effects of preoperative serum estradiol level in postmenopausal breast cancer. BMC Cancer.

[24] Zhou K., Sun P., Zhang Y., You X., Li P., Wang T. (2016). Estrogen stimulated migration and invasion of estrogen receptor negative breast cancer cells involves ezrin-dependent cross talk between G protein-coupled receptor 30 and estrogen receptor beta signaling. Steroids.

[25] Gupta P.B., Proia D., Cingoz O., Weremowics J., Naber S.P., Weinberg R.A., Kuperwasser C. (2007). Systemic stromal effects of estrogen promote the growth of estrogen receptor negative cancers. Cancer Res..

[26] Lange C.A., Yee D. (2008). Progesterone and breast cancer. Women’s Health.

[27] Rossmanith W.G., Scherbaum W.A., Lauritzen C. (1991). Gonadotropin secretion during aging in postmenopausal women. Neuroendocrinology.

[28] Siraj A., Desestret V., Antoine M., Fromont G., Huerre M., Sanson M. (2013). Expression of FSHR by the vascular endothelium in tumour metastasis. BMC Cancer.

[29] Rojas L.V., Nieves K., Suarez E., Ortiz A.P., Rivera A., Romaguera J. (2008). Thyroid-stimulating hormone and follicle-stimulating hormone status in Hispanic women during the menopause transition. Ethn. Dis..

[30] Tanaka Y., Kuwabara K., Okazaki T., Fujita T., Oizumi I., Kaiho S., Ogata E. (2000). Gonadotropins stimulate growth of MCF-7 human breast cancer cells by promoting intracellular conversion of adrenal androgen to estrogens. Oncology.

[31] Zhou J., Chen Y., Huang Y., Long J., Wan F., Zhan S. (2013). Serum follicle stimulating hormone level is associated with human epidermal growth factor receptor type 2 and Ki67 expression in postmenopausal females with breast cancer. Oncol. Lett..

[32] Arvold N.D., Taghian A.G., Niemierko A., Abi Raad R.F., Sreedhara M., Nguyen P.L., Bellon J.R., Wong J.S., Smith B.L., Harris J.R. (2011). Age, Breast Cancer Subtype Approximation, and Local Recurrence After Breast-Conserving Therapy. J. Clin. Oncol..

[33] Voduc K.D., Cheang M.C., Tyldesley S., Gelmon K., Nielsen T.O., Kennecke H. (2010). Breast cancer subtypes and the risk of local and regional relapse. J. Clin. Oncol..

[34] Dixon J.M. (2014). Endocrine Resistance in Breast Cancer. New J. Sci..

[35] Mourouzis I., Tzovaras A., Armonis B., Ardavanis A., Skondra M., Misitzis J., Pectasides D., Pantos C. (2015). Are thyroid hormone and tumor cell proliferation in human breast cancers positive for HER2 associated?. Int. J. Endocrinol..

[36] Ali A., Mir M., Bashir S., Hassan T. (2011). Impact of serum thyroid hormones and estrogen status on the risk of breast cancer in Kashmiri women. J. Cell Sci. Ther..

[37] Ying S., Myers K., Bottomley S., Helleday T., Bryant H.E. (2009). BRCA2- dependent homologous recombination is required for repair of Arsenite-induced replication lesions in mammalian cells. Nucleic Acid Res..

[38] Xu Y., Tokar E.J., Waalkes M.P. (2014). Arsenic-induced cancer cell phenotype in human breast epithelia is estrogen receptor-independent but involves aromatase activation. Arch. Toxicol..

[39] Davey J.C., Nomikos A.P., Wungjiranirun M., Sherman J.R., Ingram L., Batki C., Lariviere J.P., Hamilton J.W. (2008). Arsenic as an endocrine disruptor: Arsenic disrupts retinoic acid receptor and thyroid hormone receptor-mediated gene regulation and thyroid hormone mediated amphibian tail metamorphosis. Environ. Health Perspect..

[40] Sun H.J., Xiang P., Luo J., Hong H., Lin H., Li H.B., Ma L.Q. (2016). Mechanisms of arsenic disruption on gonadal, adrenal and thyroid endocrine systems in humans: A review. Environ. Int..

[41] Klein D., Wan Y.J., Kamyab S., Okuda H., Sokol R.Z. (1994). Effects of toxic levels of lead on gene regulation in the male axis: Increase in mRNA and intracellular stores of gonadotrophs within the central nervous system. Biol. Reprod..

